# Editorial: Organelle Autophagy in Plant Development

**DOI:** 10.3389/fpls.2020.00502

**Published:** 2020-05-05

**Authors:** Masanori Izumi, Kohki Yoshimoto, Henri Batoko

**Affiliations:** ^1^Center for Sustainable Resource Science, RIKEN, Wako, Japan; ^2^Department of Life Sciences, School of Agriculture, Meiji University, Kawasaki, Japan; ^3^Louvain Institute of Biomolecular Science and Technology, University of Louvain, Louvain-la-Neuve, Belgium

**Keywords:** organelles, autophagy, plant development, selective autophagy, carbon metabolism, autophagy receptors

To maintain cellular homeostasis, eukaryotes must control the function, quality and quantity of organelles through organelle degradation. Autophagy is an evolutionarily conserved process leading to the selective degradation of specific organelles in the vacuole/lysosomes. The typical autophagy process is macroautophagy, during which the nascent double-membrane-bound vesicles termed autophagosomes engulf target organelles and transport them into the vacuole/lysosomes. Studies in budding yeast *Saccharomyces cerevisiae* identified the core AUTOPHAGY related (Atg) proteins (Atg1-10, Atg12-14, Atg16, Atg18) that are required for the autophagosome formation. Microautophagy displays a distinct type of membrane dynamics in which vacuolar/lysosomal membrane assists the sequestering of a portion of cytoplasmic constituents and target organelles. Compared to macroautophagy, the molecular basis of microautophagy are relatively less clear. This Research Topic aimed at summarizing the recent advances on the roles of selective autophagy for plant organelles during plant development.

Goto-Yamada et al. found that microautophagy occurs in Arabidopsis root cells and Tobacco BY2 cultured cells. When Arabidopsis roots or BY2 cells are exposed to sugar starvation, probe-labeled tonoplast invaginates and the resulting vesicles accumulate in the vacuolar lumen. Inactivation of ATG5 or ATG7 suppresses this microautophagy process. The light microscopy observations suggest that in these cell types, microautophagy specifically degrades vacuolar membrane itself. Additionally, in BY2 cells, microautophagy might specifically capture cytoplasm-localized, acidic vesicles that are produced in response to starvation (Goto-Yamada et al). Thus, microautophagy might contribute to degrade such organelles during sugar starvation.

In rice, autophagy is essential for pollen development. Hanamata et al. performed a comprehensive RNA-sequencing analysis that assesses the changes of transcript profiles during pollen development in wild-type and autophagy-deficient *atg7* rice. This study showed that genes involved in programmed cell death within the tapetum, in organelles or carbon/lipid metabolism are transiently upregulated during the development of wild-type pollens, but partially delayed in the *atg7* mutant. From these data the authors inferred that autophagy might control gene expression and organelle quality during tapetal programmed cell death for pollen maturation (Hanamata et al).

Norizuki et al. developed a model system for autophagy study in liverwort *Marchantia polymorpha*. The Marchantia genome contains most of the core *ATG* genes. However, in comparison to Arabidopsis that has nine *ATG8* paralogues (*AtATG8a to i*), Marchantia has just two *ATG8s* (*MpATG8a* and *b*). Such a low potential functional redundancy in *ATG* genes is more appealing for genetic analysis. Expression of fluorescent protein-tagged MpATG8s resulted in visualization of vacuolar accumulation of autophagic vesicles in this system. Using genome editing to inactivate some *ATG* genes and the aforementioned tool allow the authors to identify some critical genes which activity is required for autophagic vesicle accumulation in the vacuolar lumen. Thus, the Marchantia model could be a simple and strong system to elucidate the molecular mechanism of autophagy including selective organelle degradation.

The current Research Topic also includes five mini-reviews that summarize the relationship between plant autophagy and lipids metabolism (Yoshitake et al.; Kajikawa and Fukuzawa), peroxisome biology (Olmedilla and Sandalio), endoplasmic reticulum (ER), (Zeng et al.) and mitochondrial homeostasis (Furukawa et al.).

Algae and plant cells accumulate storage lipids, triacylglycerols (TAGs), in response to environmental changes such as carbon or nitrogen starvation. Yoshitake et al. discussed the roles of autophagy in metabolisms of storage lipids in algae and plants. Kajikawa and Fukuzawa also assessed involvements of autophagy in carbon and lipid metabolisms based on the recent identification of core *atg* mutants in the green algae *Chlamydomonas reinhardii*. In algae, the degradation of TAGs accumulated as a result of nitrogen starvation is suppressed in *atg8* mutants (Kajikawa and Fukuzawa). Although the main route for TAGs degradation is β-oxidation within peroxisomes in plants, recent reports showed the impact of autophagy deficiency on the lipid metabolism in Arabidopsis hypocotyls, and rice pollens (Yoshitake et al., Hanamata et al). Thus, these mini-reviews suggest a potential role for selective autophagy or lipophagy in lipid metabolism in alga and plants. The direct visualization of autophagic transports of lipid droplets would help advance our understanding of this degradation pathway and its potential role in plant/algae physiology and development.

Peroxisomes are well-characterized targets of selective autophagy in yeasts. Olmedilla and Sandalio summarized the current understanding of peroxisome autophagy (pexophagy) in Arabidopsis, and its possible regulatory mechanisms. Although many researchers have focused on the autophagic receptors that act as bridge between the cargo and the autophagosomal membrane, pexophagy receptors have not yet been characterized in plants. The authors discussed the involvement of PEROXINs (PEXs) in pexophagy induction.

Secretory proteins are translated and properly folded within the ER. Although the misfolded proteins can be rapidly degraded through 26S proteasomal system, the accumulation of misfolded proteins due to environmental stresses or to treatments with chemicals such as tunicamycin or dithiothreitol causes severe ER stress. Zeng et al. discussed the relationships between ER autophagy (ER-phagy or reticulophagy) and the ER-stress responses. The ER stress induces ER-phagy in plant cells. However, the recognition mechanism of ER structures by autophagosomes remains unclear. Plant genomes encode putative conserved homologs of ER-phagy receptors described in mammals. It is tempting to speculate that the plant-related ER-phagy receptors could play similar roles as they do in mammalian cells (Zeng et al).

Selective autophagic degradation of mitochondria termed mitophagy has been widely studies in yeast and mammals. Furukawa et al. summarized the molecular mechanism of mitophagy in yeast and mammals and contrast these findings with the relevant information regarding this pathway in the plant. Although plant homologs of mammalian or yeast mitophagy receptors have not been found, some computational analyses suggest possible ATG8-interacting proteins on the plant mitochondrial outer membrane (Furukawa et al). It would be interesting to experimentally confirm this possibility in the near future.

Over the last decade, numerous significant studies have characterized selective degradation of specific organelles in plant cells through the autophagic pathway. It is becoming clear that autophagic degradation of organelle is essential for plant growth, stress adaptations and productivity. However, the molecular mechanisms at the basis of this critical pathway including the receptor proteins that confer target selectivity remain to be elucidated. This would require some out-of-the-box approaches as most of the known selective autophagy receptors appear not to be evolutionarily conserved in eukaryotes from different lineage. Interestingly, some structural and functional comparative studies (Norizuki et al; Olmedilla and Sandalio; Zeng et al; Furukawa et al.) could provide working hypotheses. Overcoming these challenges would help understanding the roles of organelle autophagy in carbon or nitrogen metabolism in plants and algae and answer some basic biological questions raised in the current Research topic (Goto-Yamada et al; Hanamata et al.; Yoshitake et al; Kajikawa and Fukuzawa).

## Author Contributions

MI prepared the first draft of this editorial. KY and HB revised the editorial. All authors approved the editorial for publication.

## Conflict of Interest

The authors declare that the research was conducted in the absence of any commercial or financial relationships that could be construed as a potential conflict of interest.

